# The origin, deployment, and evolution of a plant-parasitic nematode effectorome

**DOI:** 10.1371/journal.ppat.1012395

**Published:** 2024-07-29

**Authors:** Beth Molloy, Dio S. Shin, Jonathan Long, Clement Pellegrin, Beatrice Senatori, Paulo Vieira, Peter J. Thorpe, Anika Damm, Mariam Ahmad, Kerry Vermeulen, Lida Derevnina, Siyuan Wei, Alexis Sperling, Estefany Reyes Estévez, Samuel Bruty, Victor Hugo Moura de Souza, Olaf Prosper Kranse, Tom Maier, Thomas Baum, Sebastian Eves-van den Akker

**Affiliations:** 1 The Crop Science Centre, Department of Plant Sciences, University of Cambridge, Cambridge, United Kingdom; 2 Mycology and Nematology Genetic Diversity and Biology Laboratory, United States Department of Agriculture—Agricultural Research Service, Beltsville, Maryland, United States of America; 3 The Data Analysis Group, School of Life Sciences, University of Dundee, Dundee, Scotland, United Kingdom; 4 Department of Plant Pathology, Entomology and Microbiology, Iowa State University, Ames, Iowa, United States of America; University of Cologne, GERMANY

## Abstract

Plant-parasitic nematodes constrain global food security. During parasitism, they secrete effectors into the host plant from two types of pharyngeal gland cells. These effectors elicit profound changes in host biology to suppress immunity and establish a unique feeding organ from which the nematode draws nutrition. Despite the importance of effectors in nematode parasitism, there has been no comprehensive identification and characterisation of the effector repertoire of any plant-parasitic nematode. To address this, we advance techniques for gland cell isolation and transcriptional analysis to define a stringent annotation of putative effectors for the cyst nematode *Heterodera schachtii* at three key life-stages. We define 717 effector gene loci: 269 “known” high-confidence homologs of plant-parasitic nematode effectors, and 448 “novel” effectors with high gland cell expression. In doing so we define the most comprehensive “effectorome” of a plant-parasitic nematode to date. Using this effector definition, we provide the first systems-level understanding of the origin, deployment and evolution of a plant-parasitic nematode effectorome. The robust identification of the effector repertoire of a plant-parasitic nematode will underpin our understanding of nematode pathology, and hence, inform strategies for crop protection.

## Author summary

Pathogenic organisms deliver so called “effectors”, typically proteins, into their host to alter cell structure and function. Identifying and characterising the repertoire of effector-encoding genes is requisite to understanding infection. Here we advance methods and understanding, in combination with an extensive literature survey, to define a stringent annotation of putative effectors for the plant-parasitic cyst nematode, *Heterodera schachtii*. Using this effector definition, we provide the first systems-level understanding of the origin, deployment, and evolution of a plant-parasitic nematode effectorome.

Given that plant-parasitic nematodes are major, and in some cases dominant, threats to global food security, it is widely cited that understanding how they infect their host (i.e. effectors) will deliver routes to alleviate their threat to agriculture.

## Introduction

Plant-parasitic nematodes (PPNs) are devastating crop parasites which present a considerable threat to global food security. Every major crop can be parasitised by at least one species of nematode, with collective damage estimated at over 80 billion dollars worldwide [1]. The most damaging nematode species are those capable of forming intimate, long-term biotrophic relationships with their host plant–namely, the cyst (genera *Heterodera* and *Globodera*) and root-knot nematodes (genus *Meloidogyne*) [2]. These species are capable of eliciting profound changes in plant biology to form a unique pseudo-organ: a feeding site from which the nematode draws all its nutrition for the remainder of its life cycle [3]. Through this pseudo-organ, the nematode drains the host of essential nutrients and water. This directly affects the fitness of the host and, in the rarest of cases, can result in the death of the plant.

Upon nematode infection, the extent of changes to the plant are vast: nematodes have evolved to suppress the plant immune system [4,5], and to form a feeding site by manipulating host cell biology, physiology and development [6]. In cyst nematodes, this includes the arrest of the plant cell cycle in G2 phase, the fragmentation of the vacuole, the proliferation of numerous organelles (including the smooth endoplasmic reticulum, ribosomes, mitochondria, and plastids), and the dissolution of the cell wall leading to the fusion of hundreds of adjacent cells to form a syncytial feeding organ [7,8]. In this way, a nutrient sink is formed to sustain the female nematode as it matures and reproduces.

To achieve these changes, nematodes–like other parasites and pathogens–deploy molecular tools called effectors. Effectors can be described as parasite or pathogen-secreted molecules that alter host-cell structure and function, often to the benefit of the pathogen [9]. Crucially, in PPNs, these effectors are almost exclusively secreted from two types of specialised pharyngeal gland cells: the subventral glands (SvGs) and the dorsal gland (DG) [10]. Expression of secreted proteins in the gland cells is therefore considered synonymous with proteinaceous effector definition in these species. Changes in the content and activity of the gland cells suggests that the SvGs may be predominantly responsible for effector secretion during the early life-stages, whereas the DG is predominantly active during the sedentary life-stages [10,11].

Despite the clear importance of nematode effectors in feeding site establishment, and hence in the success of nematode pathology, we still have an incomplete understanding of the biological functions, or even identities, of individual effectors [6]. As is the case for plant pathology in general, predicting genes encoding effectors represents a major bottleneck for the field [12].

One property that effectors hold in common is that they are secreted by the pathogen into the host. As a result, the presence of a secretion signal, and the absence of transmembrane domains or ER-retention signals have been the dominant criteria for the prediction of proteinaceous effectors in eukaryotic (and many bacterial) plant pathogens [13,14]. However, differentiating the subset of secreted proteins which actually function as effectors from the full set of pathogen secreted proteins is a perennial challenge. For some plant pathogens, characteristic sequences (e.g. RxLR oomycete effectors [15]), structural motifs (e.g. the MAX (*Magnaporthe* Avrs and ToxB like) fold of ascomycete fungal pathogens [16]), or even codon usage bias (e.g. positive bias for -AA ending codons in unconventionally secreted cytoplasmic effectors in *Magnaporthe oryzae* [17]) can be used as an additional criterion for effector identification.

In the case of plant-parasitic nematodes, however, we can take advantage of their unique biology to reveal effector identities—specifically, the presence of specialised gland cells for effector production. This understanding led to the discovery of the DOG-box (Dorsal Oesophageal Gland box), a 6 bp non-coding motif enriched in the promoters of dorsal gland effectors in cyst nematodes [18,19]. This discovery facilitated the prediction of a superset of putative dorsal gland effectors, a number of which were experimentally validated as dorsal gland expressed genes by *in situ* hybridisation. Subsequently, much research to identify nematode effectors has also taken a genomic approach, with a focus on DNA motifs associated with known gland-cell effector gene loci in a number of plant-parasitic nematode species [20,21,22]. This approach provides a valuable, non-generic (i.e. unlike secretion signals) criterion for identifying effectors, but it is also limited in two regards. Firstly, the use of distinct motifs by distinct species of nematode leads to a lack of generalisability across nematode species. At the same time, the short length of some motifs, such as the DOG-box, leads to a lack of specificity within the genome of a given nematode species [23].

Advances in targeted transcriptomics, pioneered by Maier et al. [24] have allowed the isolation and transcriptomic analysis of plant-parasitic nematode gland cells. Here, we take advantage of these techniques to generate gland cell specific RNA-seq. libraries for the model cyst nematode, *Heterodera schachtii*, at three key life-stages. In combination with the robust reference genome for *H*. *schachtii* [25] we used these libraries to define a stringent annotation of 717 effector gene loci: 269 “known” high-confidence homologs of plant-parasitic nematode effectors, and 448 “novel” effectors with high gland cell expression. In doing so, we defined the most comprehensive effector repertoire, or “effectorome” of a plant-parasitic nematode to date. Using this effector definition, we provide the first holistic understanding of the origin, deployment and evolution of a plant-parasitic nematode effectorome.

Robust identification of the comprehensive effector repertoire of a given plant pathogen is foundational to understanding its pathology, and hence, can inform the development of resistance to crop diseases [12]. Plant pathogen effectors are also important as a means for investigating fundamental host processes, and as promising targets for biotechnological application [26,27]. Taken together, results from this work provide an overview of cyst nematode parasitism which can form the basis of further functional studies of the effectorome, and demonstrate the utility of gland-cell transcriptomics as a method for effector discovery.

## Results

### Targeted transcriptomics of *Heterodera schachtii* gland cells

**Fig 1:**
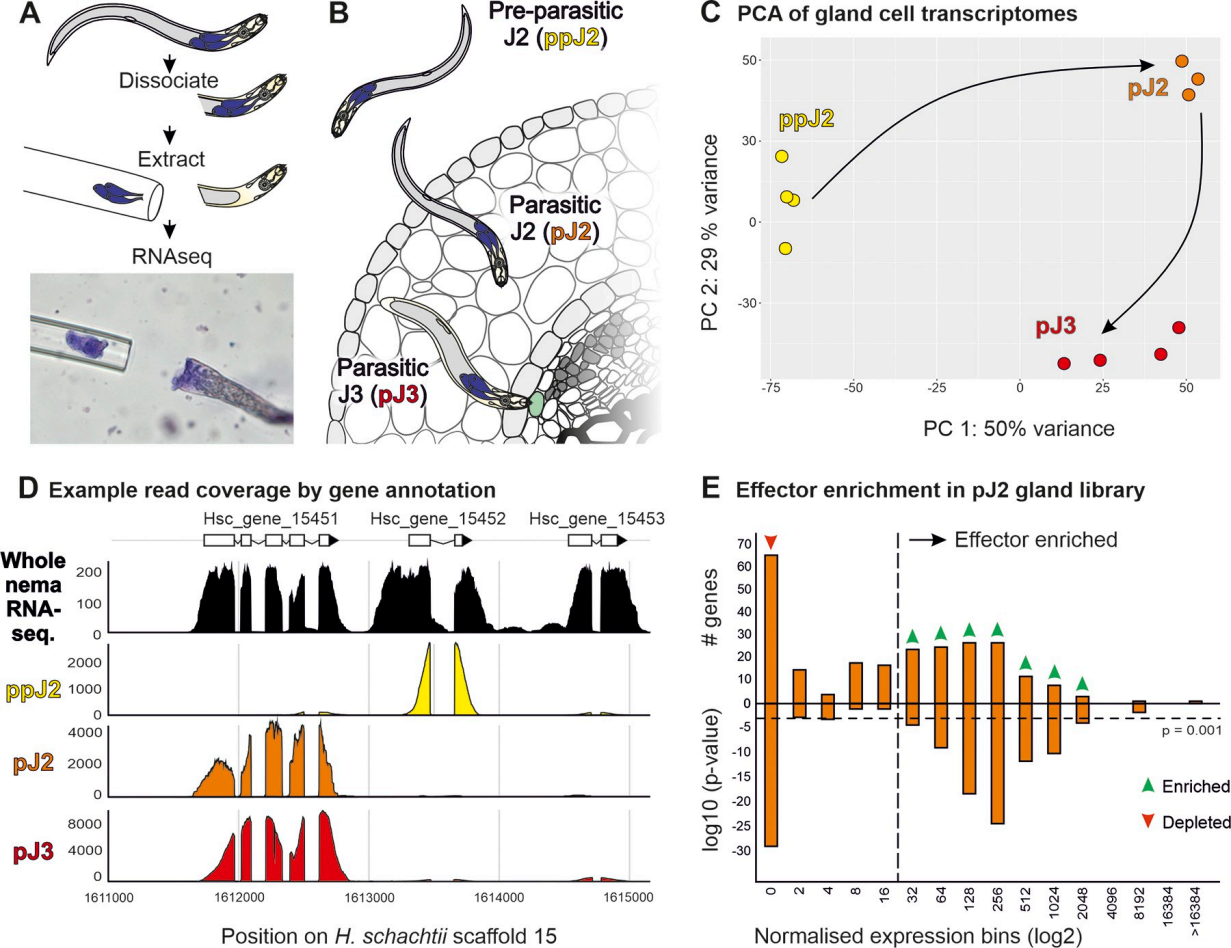
Targeted transcriptomics of *Heterodera schachtii* gland cells. **A)** Schematic representation of gland cell (blue) extraction and sequencing, with representative micrograph below. **B)** Schematic showing the establishment of cyst nematode parasitism, highlighting the three life-stages sampled in this study. **C)** Principal components 1 and 2 for gland cell expression gene read count data. Circles indicate biological replicates. Arrows indicate progression through the nematode life-stages. **D)** Example coverage of expression data for genes Hsc_gene_15451, Hsc_gene_15452 and Hsc_gene_15453 in the *H*. *schachtii* genome. The uppermost track shows gene expression data for the whole nematode (black), and the bottom three tracks show expression in gland cell libraries (pre-parasitic J2, ppJ2, yellow; parasitic J2, pJ2, orange; and parasiticJ3, pJ3, red). **E)** Effector enrichment in the parasitic J2 gland cell library (see also S1B Fig). The upper axis shows the number of effector-annotated genes in each expression bin (i.e. at each expression level). Hypergeometric distribution tests were used to determine either the enrichment (green arrows) or depletion (red arrow) of effectors in each bin. The lower axis shows the p-values from these tests. The horizontal dashed line denotes a p-value of 0.001. The vertical dashed line denotes the threshold expression level above which effector genes are consistently enriched.

Gland cells were extracted from three life-stages of *H*. *schachtii* covering the transition to biotrophy: freshly hatched pre-parasitic second-stage juveniles (ppJ2, i.e. before exposure to the host); parasitic J2s (pJ2, i.e. predominantly motile stages extracted from host tissues); and parasitic J3s (pJ3, i.e. sedentary nematodes engaged in biotrophy, Fig 1A and 1B). For each stage, mRNA from pools of gland cells was sequenced and aligned to the reference genome of *H*. *schachtii* [25].

Gland cell libraries from each life-stage are distinctly different (Fig 1C and 1D), likely capturing changes in expression of the effector repertoire during the transition to biotrophy. As expected, effector-annotated genes and genes encoding putatively secreted proteins (both as defined by Siddique et al. [25]) typically have high coverage in these libraries. However, the values for normalised read coverage per gene form a continuous distribution. To convert this continuous distribution into a binary classification (i.e. either above or below the expression level of effector-annotated genes), all genes were first assigned to one of several expression bins (increasing in a log series) based on how highly they were expressed in a given gland cell library. In general, effector-annotated genes (Fig 1E), and genes encoding putatively secreted proteins (S1A–S1C Fig), are both statistically depleted in low expression bins and statistically enriched in high expression bins (hypergeometric test, p<0.001). Enrichment of effectors validates the libraries and enrichment of many additional putative secreted proteins suggest that the known effector repertoire is non-exhaustive. Therefore, the lowest expression bin enriched in either—but in most cases both—effector-annotated genes and genes encoding putatively secreted proteins was used to define a cutoff for gland cell expression in each library. Above this cutoff, gland-expressed genes encoding putatively secreted proteins were considered putative effectors [20,28,29]. A majority (63%) of previously effector-annotated genes were recaptured above expression thresholds in gland cell libraries using this approach (i.e. 37% generally have non-zero expression values in the gland cell libraries but not above threshold). Assuming a similar percentage of novel effectors are captured above these thresholds, we can estimate that this effector repertoire is on the order of 72% complete. Gene models for all effector-annotated genes, as well as all genes encoding putatively secreted proteins and expressed above these cutoffs (725 genes in total) were manually inspected and curated in order to confirm the accuracy of the gene model and minimise false positives prior to defining a list of effectors.

### The *H*. *schachtii* effectorome

To define the effectorome of a plant-parasitic nematode, we combined putative effector identification from the targeted gland cell transcriptomics of ppJ2, pJ2, and pJ3 with effector annotation (largely from Siddique et al. [25], but updated with the latest literature). The resulting 725 gene models were manually examined and curated on Apollo [30] prior to defining a list of effectors to minimise false positives, maximise their robustness, and ultimately to create the highest quality reference database for cyst nematodes with the available data (S1 Table). To do this, we retained genes with sequence similarity to previously published effectors if they encode putatively secreted proteins, termed throughout the “knowns”, regardless of their expression in the gland cell libraries (although most are highly expressed), and augmented this with novel putative effectors defined by improbably high gland cell expression, termed the “novels”. Taken together, this combined effector set likely includes most known effectors but underestimates novel effectors (by including only those most highly expressed). Therefore, the combined putative effectorome should be considered a lower bound for a plant-parasitic cyst nematode, comprising 827 transcripts (309 known, 518 novel) from 717 individual gene loci (269 known, 448 novel)—S1 Table.

The pre-parasitic J2 gland libraries contributed the least, and the parasitic J2 gland libraries contributed the most, to effector prediction (S1F Fig). This is consistent with the expression of known effectors, which typically peak somewhere between 10 and 48 hours post infection (hpi) [25].

**Fig 2:**
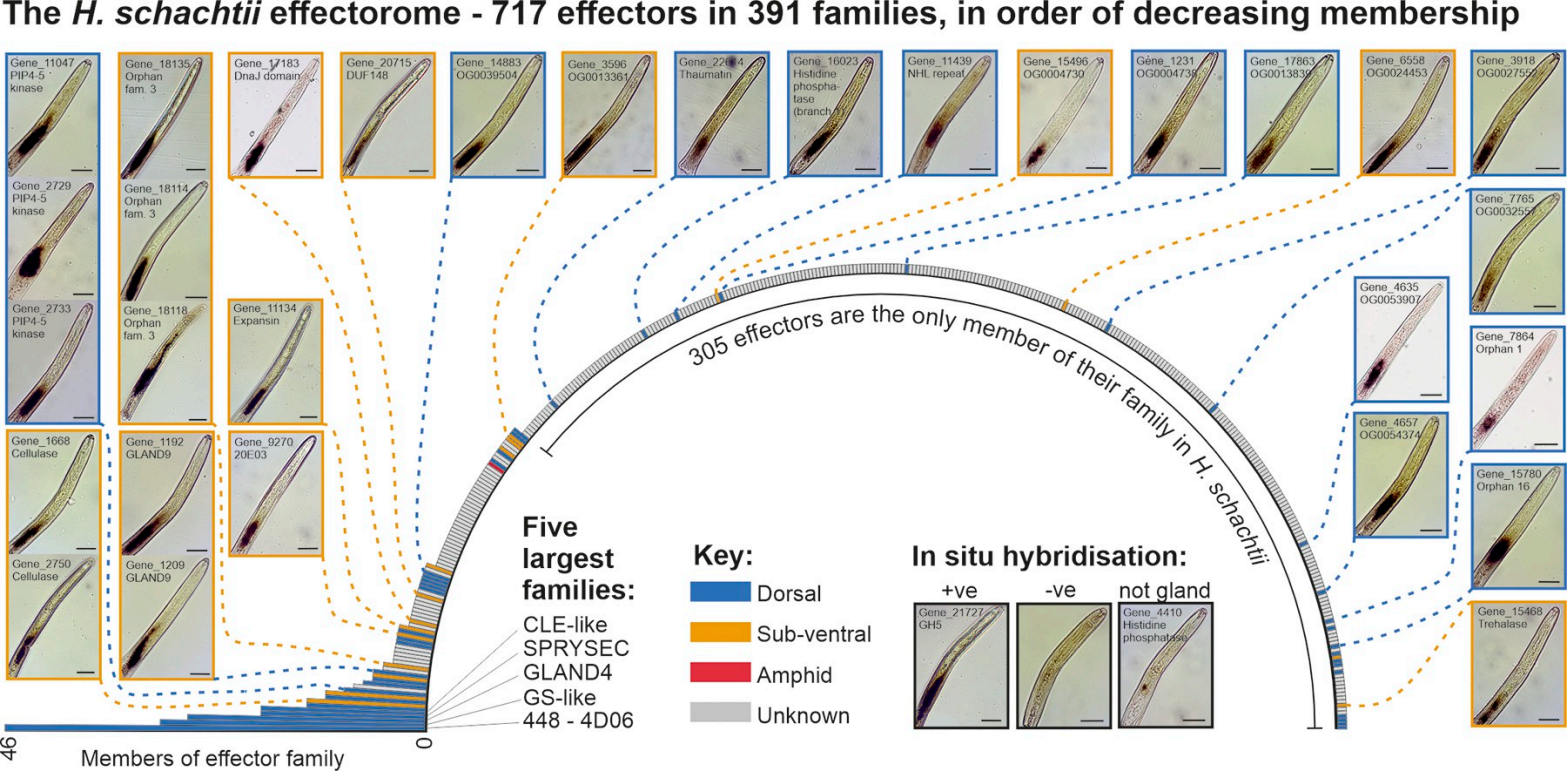
Gene families of the *Heterodera schachtii* effectorome. Radial bar chart showing the number of effectors in each of the 391 effector families, in order of decreasing membership. The five largest families are named (for additional families see S1 Table). Colours indicate gland cell expression of the family, as determined by *in situ* hybridisation, where it is known for at least one member of said family in a plant-parasitic nematode species [25]. Inset panels around the bar chart show *in situ* hybridisations produced in this study of *H*. *schachtii* effector transcripts to the gland cells. Positive (+ve), negative (-ve), and non-gland cell expression (a small unknown cell type adjacent to the glands) are shown. Scale bars represent 25 μm.

The 717 genes of the putative effectorome can be grouped into 391 gene “families” based on a combination of common Pfam domains (e.g. the GS-like effectors [31]), pre-computed OrthoMCL [32] coupled with BLAST for those effectors that do not encode known Pfam domains and cannot be assigned to an orthogroup, or expert knowledge where the nature of the effector precludes the former (e.g. CLEs [33] have neither Pfam domains nor function well in BLAST-based analyses due to their short size). The grouping of the putative effectorome into families is highly skewed (Fig 2): the 5 largest families (1.3%) contain a fifth (19%) of all effectors; in contrast, 305 families (78%) contain only one effector, and so 43% of all effectors are the only member of their family in *H*. *schachtii*. Previously, gland cell specific expression was assigned to the 248 high-confidence predicted effectors by Siddique et al. [25], where the location of expression was known for an effector in the same family in a plant-parasitic nematode. Gland cell specific expression was assigned accordingly for new predicted effector genes which were identified as members of these families in this study, or those that were validated by *in situ* hybridisation in this paper (Fig 2). Interestingly, the 5 largest gene families are all dorsal gland expressed, and contribute in part to the fact that the DG effectors numerically dominate SvG effectors in the putative effectorome by nearly 5:1 (Fig 2).

We used *in situ* hybridisation, interrogating genes across a range of families, including “knowns” and “novels”, to validate individual genes within the putative effectorome, and to some extent the effectorome as a whole. Of the 31 genes we tested, all but one were confirmed to be gland cell expressed (15 DG, 15 SvG): the exception being Hsc_gene_4410, which was expressed in a small unknown cell type adjacent to the glands, and was removed from further analyses. Taken together, these data point to the robustness of combined targeted transcriptomics, manual annotation, and the identification pipeline. With this robustly-tested and high-confidence putative effectorome in hand, we can now analyse the nature of the *H*. *schachtii* effector repertoire.

### The effector network

**Fig 3:**
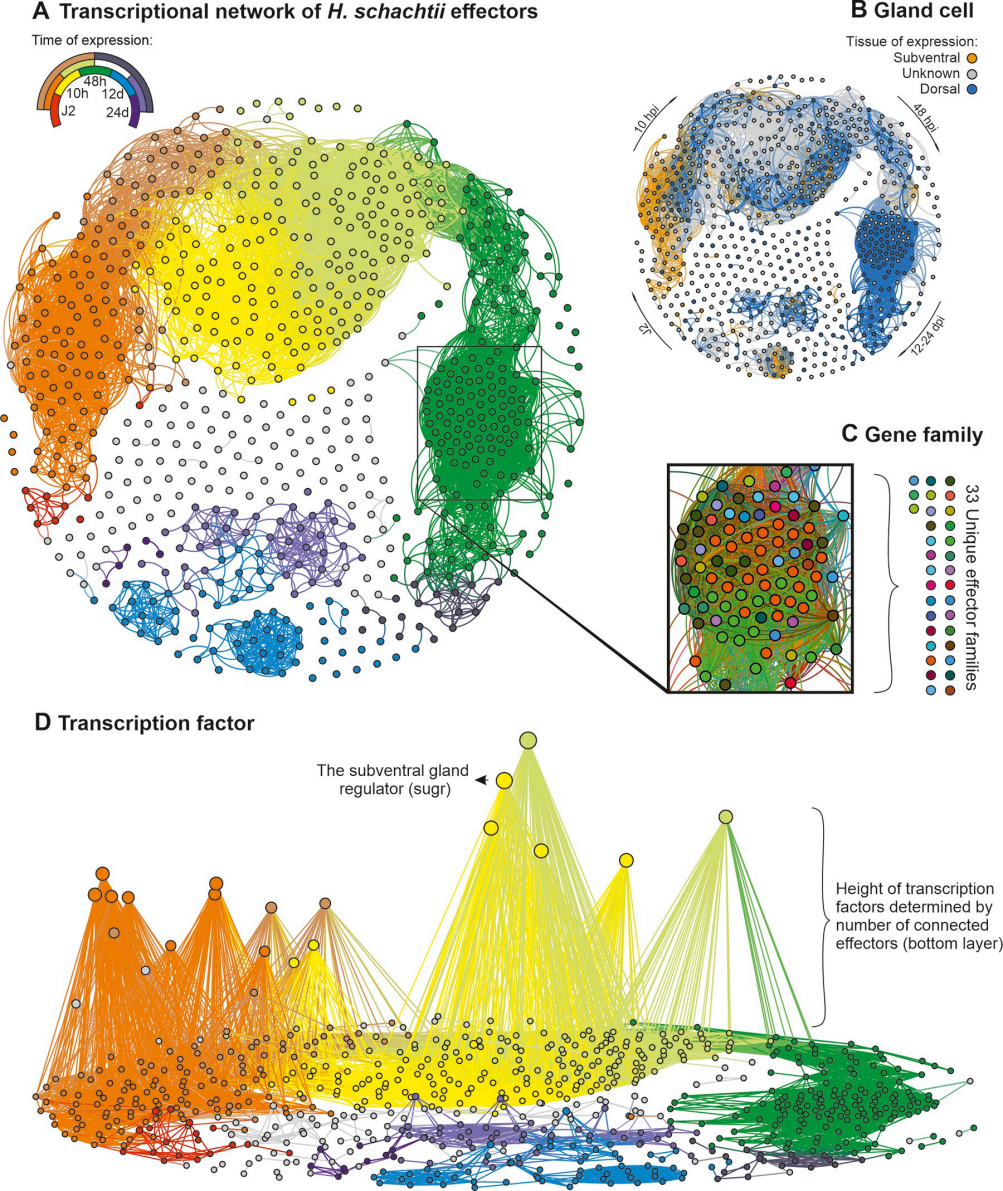
The effector network. **A)** A transcriptional network of *Heterodera schachtii* effectors. Each circle represents one effector gene locus, and connections between circles indicate a correlation in expression above 0.975 (distance correlation coefficient) across the life cycle. The key indicates the expression supercluster as defined by Siddique et al. [25]—where, for example, genes with expression peaking at J2 are shown in red, 10 hours post infection in yellow, and J2_10 hours post infection shown in orange. **B)** The same transcriptional network coloured by gland cell expression (subventral gold, and dorsal blue) of predicted effector in a given family, where this information is known for at least one effectors in that family in a plant-parasitic nematode **C)** A portion of the 48 hours post infection subcluster coloured by effector family. The key is to illustrate the number of unique families (for specific families therein, see S1 Table). **D)** The effector network re-computed with the addition of endogenous nematode transcription factors with at least two non-zero values in gland cell expression libraries and at least one connection at this threshold. Putative effectors are on the X,Y plane, and transcription factors on the Z axis (height in Z is determined by the number of connections with effectors, with the most connected TFs appearing higher in Z). Networks are available in DRYAD repository 10.5061/dryad.rfj6q57hn.

By cross referencing the putative effectorome with the life-stage specific transcriptome [25] we were able to generate a transcriptional network of effectors that elegantly describes the progression of parasitism. Nodes (defined by the 717 putative effector gene loci), are connected by 10,866 edges (defined by concerted expression across the life cycle above an arbitrary threshold distance correlation coefficient of 0.975), to reveal a highly connected network (markedly different to a control network of 717 random genes in the genome, S2 Fig). Remarkably, all of the connections in the network represent strong positive correlations between effectors. Most effectors have a connection (607/717), and on average are connected to 30 others. This results in one large supercluster, containing 66% of all effectors, connecting those exclusively expressed at pre-parasitic J2 right through to those exclusively expressed during sustained biotrophy (defined as 48 hours post infection to 24 days post infection inclusive (dpi)), by a series of linked subclusters describing the stages in between (Fig 3A). Two smaller superclusters contain 9% and 3% of effectors, principally those expressed at various individual times between 12 days and 24 dpi. The remaining 22% of effectors are largely independent in the network.

We can map various attributes of the effectorome onto the effector network to interrogate both the network itself, and the nature of this parasitism as a whole. For example, we can map expression of effectors in specific gland cell types for effectors in a given family, where this information is known for at least one member of a putative effector family in a plant-parasitic nematode. Mapping gland cell expression to the network in this way supports the accepted view that the subventral gland precedes the dorsal gland during infection: known effectors in the J2 subcluster are exclusively subventral, and known effectors in the 48 hpi subcluster are almost exclusively dorsal (Fig 3B). Given that some times of infection are dominated by a particular gland, this would in principle allow prediction of spatial expression from the network in some (albeit a minority of) cases.

Mapping gene families onto the network reveals the intuitive finding that many effector families are co-expressed in time. However, in some cases, effector co-expression is more similar between families than within, resulting in individual subclusters that describe a given time being assembled from a diversity of effector families. For example, examining a particularly highly connected part of the 48 hpi subcluster reveals 88 effectors from 33 unique families (Fig 3C).

To understand how effectors from unrelated families are transcriptionally regulated in such a concerted manner, correlations in life cycle expression between putative effectors and endogenous transcription factors were computed (Fig 3D). Of the 376 transcription factors predicted in the *H*. *schachtii* genome, 238 show more than one non-zero value in the gland cell expression data. Of those, 99 are connected to effectors in the network with a threshold distance correlation coefficient above 0.975 (Fig 3D). Ranking transcription factors in the network by the number of connections they have with putative effectors independently highlights the subventral gland regulator (sugr), a transcription factor that has been shown to regulate the transcription of genes encoding subventral gland effectors, as the second most connected transcription factor [34]. Additional highly connected transcription factors likely regulate other parts of the effector network, and are also highlighted by this approach.

### Evolutionary origins of effectors

**Fig 4:**
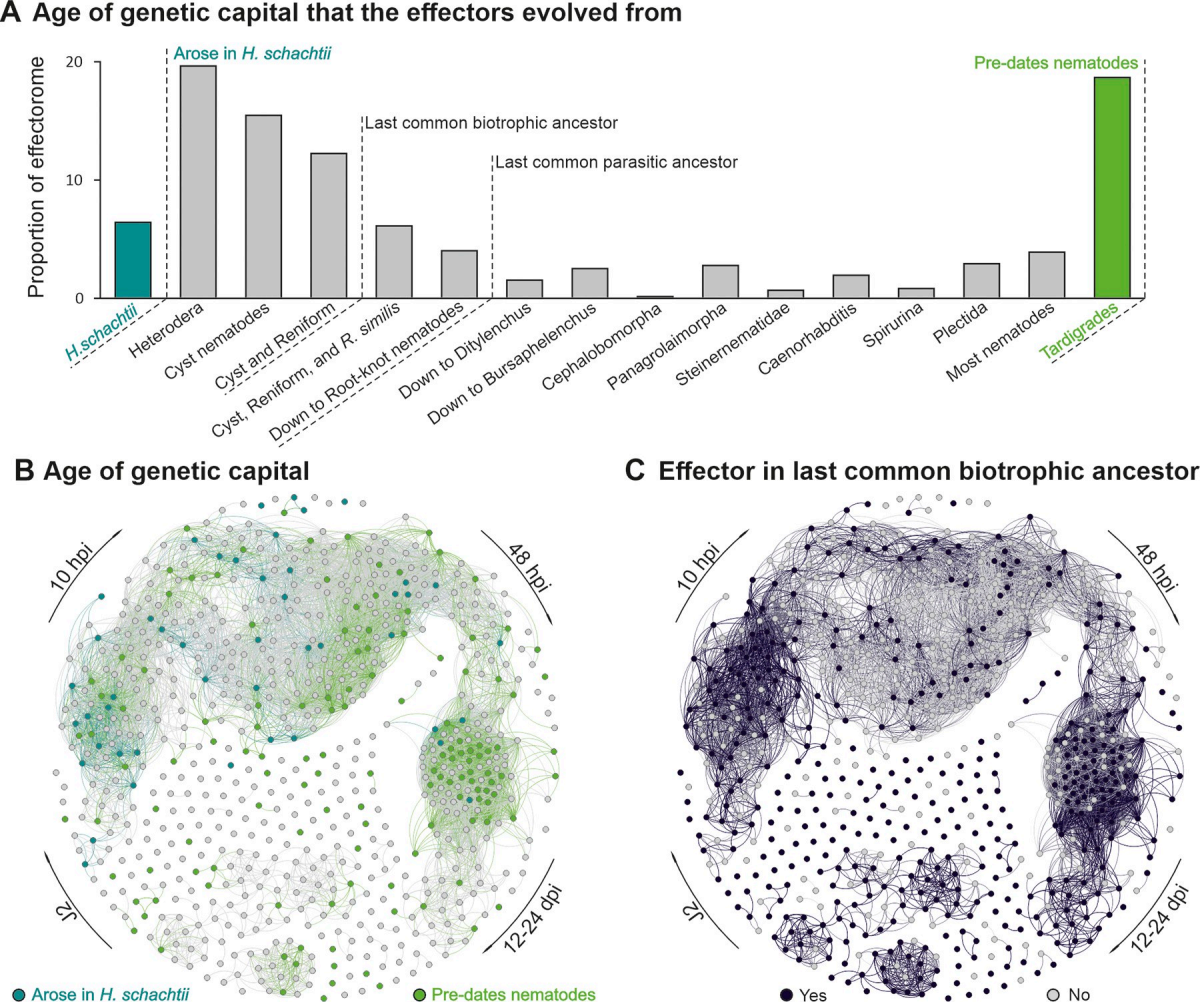
Evolutionary origins of effectors. **A**) A frequency distribution of age of genetic capital (i.e. gene sequence) from which putative effectors evolved (i.e. the most distant relative with a similar sequence to a given effector). Two categories are highlighted: those exclusive to *H*. *schachtii* (teal) and those that pre-date the phylum Nematoda (green). **B**) Superposition onto the network of genes exclusive to *H*. *schachtii* and gene pre-dating Nematoda **C**) Whether or not each effector is present in an orthogroup with a putative secreted protein from any *Globodera* spp.

To determine when and how effectors evolve, we first cross referenced the putative effectorome with orthologous gene clustering of 61 species, covering the breadth of the nematode phylum and including two outgroup taxa [32]. Effector families were then classified by when the genetic capital (i.e. the underlying genomic sequence) that gave rise to the family is first observed in the phylum according to this orthogroup analysis. By doing this, two broad classes were identified as key contributors to the effectorome (Fig 4A): 1) approximately 19% of effectors (11% of families) are sequence similar to highly conserved genes that predate the nematode phylum (i.e. have a similar sequence in the Tardigrade outgroup), and likely represent duplication and subsequent neofunctionalization (as in the GS-like effectors [31], SPRYSECs [35], peptidases [36], etc.); and 2) approximately 54% of effectors (59% of families) are only sequence similar to genes that arose since the last common biotrophic ancestor with *Rotylenchulus reniformis*. Very few effectors, approximately 6%, have no similar sequence in any other organism, even in the close sister species *Heterodera glycines*, and are here termed “orphan” effectors.

Contrary to the apparent emerging trend (e.g. 6% of fungal effectors are MAX effectors), we find no evidence of a conserved effector fold, for those effectors that can be predicted at present. We computationally predicted the structures of all proteins in *H*. *schachtii* and *Globodera rostochiensis* (all models deposited under DRYAD repository 10.5061/dryad.rfj6q57hn). Most effectors are sequence dissimilar to characterised proteins and so result in low-confidence fold prediction. Of the 827 putative effector transcripts identified in this study, 335 transcripts from 314 gene loci fold with an average pLDDT > 50 and pTM > 0.5. A structural similarity network (built using structure-based BLAST, Foldseek [37]) does not identify groups of effectors that would not have otherwise been grouped by sequence similarity and/or expert knowledge of characteristic effector motifs (S3 Fig). Using this approach did, however, did reveal unambiguous “hybrid” effectors (S4 Fig) resulting from the fusion of two different effector domains, although these hybrid effectors could also have been identifiable using sequence similarity alone.

These data demonstrate that the effectorome of *H*. *schachtii* is assembled from a diversity of genetic capital that itself arose over an extremely long period of time. It does not show when an actual effector was assembled from said capital. We therefore sought to determine which of the *H*. *schachtii* effector gene families were likely already present as effectors in the last common ancestor of the cyst nematodes, circa 100 million years ago. We identified orthogroups that contain putative *H*. *schachtii* effectors, and determined whether the corresponding members of those orthogroups in either *Globodera pallida* or *G*. *rostochiensis* also encode putatively secreted proteins. Using this rough proxy, we estimate that a majority (58%) of effectors were likely present in the last common ancestor of the cyst nematodes (Fig 4C).

### Cross-kingdom gene regulatory network

**Fig 5:**
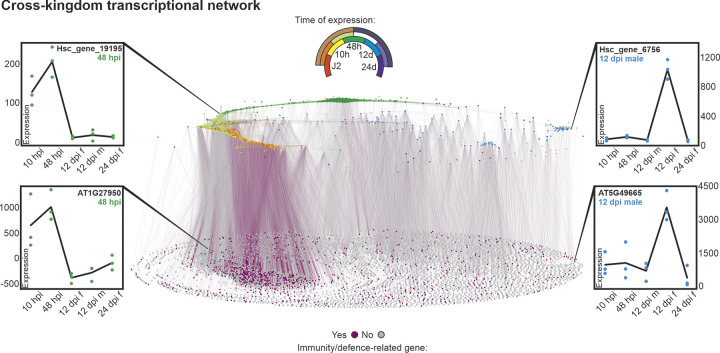
The cross-kingdom transcriptional network. A transcriptional network of *H*. *schachtii* effector genes and *A*. *thaliana* genes. In the upper plane, each circle represents one effector locus. In the lower plane, each circle represents a host plant gene. Connections between circles indicate a correlation in expression above 0.975 (distance correlation coefficient) across infection. The upper key indicates the expression supercluster for effectors as defined by Siddique et al. [25] and corresponds to the effector plane only. The lower key indicates whether a host plant gene was included in our immune/defence-related gene dataset (purple) or not (grey), and corresponds to the plant genes plane only. Expression profiles (left and right) show gene expression for nematode effectors (top) and plant genes (bottom) with highly correlated expression patterns across the infective life-stages. Individual data points represent biological replicates. Lines represent mean expression across biological replicates. Networks are available in DRAYD repository 10.5061/dryad.rfj6q57hn.

To determine which genes in the host are transcriptionally co-regulated with nematode effectors, we compared life-stage specific transcriptomic data for the putative nematode effectors and the host plant, *Arabidopsis thaliana* [25]. From this we connected each node in the effector network to the host plant genes with correlated expression profiles (defined by concerted expression across infection with a threshold distance correlation coefficient above 0.975). This yielded a network with 7,000 nodes (715 effectors and 6,285 plant genes) and 201,711 edges (28,270 effector-effector connections and 173,441 effector-plant gene connections, Fig 5). The number of connections between effectors and plant genes shows an extremely skewed distribution. The most highly connected effector, a putative effector peaking at 10 hpi and assigned to the gene family OG0038700, is connected to 1,054 plant genes (with largely overlapping identities), whilst 50% of effectors are connected to 125 plant genes or fewer. The distribution of connections between plant genes and effectors is similarly skewed, with the three most highly connected plant genes each connected to 163 effectors, 65.2% of plant genes connected to 10 effectors or fewer, and 23.3% of plant genes connected to just 1 effector.

To understand how effectors might control and/or contribute to host biological processes, for example immunity over the course of infection, we developed a dataset for plant genes present in the cross-kingdom network (i.e. highly correlated in expression with nematode effectors across infection) that are involved in immunity or defence responses. In doing so, we defined an immunity/defence-related gene as: a gene annotated with a GO term in both the GO Slim categories “response to biotic stimulus” (GO:0009607) and “response to stress” (GO:0006950) (according to GO Slim Classification for Plants [38]); or a gene annotated with the GO term “response to wounding” (GO:0009611); or a gene annotated with any other GO term with a name containing the phrases “defence response” or “immune”. In total this dataset contained 991 genes, representing 15.8% of plant genes in the network and 11.8% of all differentially expressed plant genes across the life cycle. To determine when, in the course of the infection, immunity/defence-related genes are coregulated with effectors, we compared enrichment/depletion of these genes in each of the 29 expression superclusters defined by Siddique et al. [25]. The number of connections between immunity/defence-related genes in the network and effectors in a given supercluster was enriched (1,000 bootstrap, 95% confidence intervals) mainly in superclusters containing motile stages (i.e. J2 and 10 hpi): mainly in superclusters containing motile stages (i.e. J2 and 10 hpi): “cyst_J2”, “cyst_J2_10hpi_48hpi”, “J2”, “J2_10hpi”, “10hpi” as well as the “48hpi_12dpifem._12dpimale_24dpi” and “not clustered but differentially expressed” superclusters; and depleted in superclusters containing sedentary life-stages (i.e. 12 and 24 dpi): “10hpi_12dpimale”, “12dpifem.”, “12dpifem._12dpimale”, “12dpifem._24dpi”, “12dpifem._12dmale_24dpi” and “increasing”.

Unlike the effector-effector edges, which represent exclusively positive correlations, 24.5% of effector-plant edges (42,555) are negative correlations. When the “direction” (positive or negative) of effector-plant edges is visualised in the network, positive correlations appear enriched early in infection and evenly distributed at the later life-stages. The vast majority (90.3%) of connections between the effectors and immune/defence-related genes represent positive correlations. These data provide a platform for hypothesis generation, ultimately to accelerate the interrogation of complex plant-nematode interactions.

## Discussion

### Effector identification

Through a combination of targeted transcriptomics and an extensive literature search—both coupled to manual annotation and curation—we have defined the most comprehensive putative effectorome of a plant-parasitic nematode to date. The goal was to retain genes with sequence similarity to previously published effectors, if they encode putative secreted proteins, regardless of their expression in the gland cell libraries (termed the “knowns”), and augment this with novel putative effectors defined by improbably high gland cell expression, (termed the “novels”). While we have been conservative in effector prediction, the number of effectors identified is very large: 717. Using *in situ* hybridisation to test the putative effector prediction resulted in an extremely high true positive rate (30/31 tested effector genes). Coupled with the fact that most (63%), but not all, “known” effectors were above the gland cell transcriptomics threshold, this number is likely a conservative underestimate. If we assume a similar percentage of the novels are captured above these thresholds, we can estimate that this effector repertoire is around 70% complete. To put this number in context, just under one in three genes in the secretome, and one in thirty genes in the genome (2,669 genes, and 26,739 genes respectively [25]) are likely effectors.

Despite progress in identifying promoter motifs associated with gland cell expression (i.e. the DOG Box [19]), gland cell targeted transcriptomics appears to be the most efficacious method for effector identification to date. The time of gland cell extraction has a very large impact on the number and type of effector identified. The dominant majority of putative effectors identified in this study were identified from the parasitic J2 gland cell library—even if they peak in expression at very different times of infection across the network. The parasitic J3 was the next most informative library, followed by the pre-parasitic J2 which was the least informative (almost uninformative) (S1D Fig). When we map those putative effectors identified from gland cell libraries at each life-stage onto the network, we can see, as we might expected, that the pre-parasitic J2 libraries identified effectors expressed earlier in infection and parasitic J3 libraries identify effectors later in infection, with parasitic J2 identified effectors spanning the network (S5 Fig).

Taken together, these data inform future efforts to define effectoromes of plant-parasitic nematodes: gland cell sequencing of parasitic J2 and J3 life-stages, coupled with expression-based effector enrichment analyses.

### Deployment

There are three superclusters in the transcriptional network, and at this, albeit arbitrary, threshold they are not connected: one very large supercluster that links effectors expressed at the earliest time point through to those expressed at the latest; and a two much smaller superclusters that principally contain effectors expressed at various stages 12 dpi onwards. This gap in connectedness most likely reflects the relatively large gap in time between two measurement points (48 hpi and 12 dpi) and not a true biological phenomenon.

Nevertheless, the largest supercluster elegantly highlights the deployment of effectors over time. As previously noted [25] almost no effectors peak in expression at the pre-parasitic J2 stage. There is then a bulge centred on 10 hpi, followed by a second separate bulge at 48 hpi. These data, and indeed the identification of different effectors from each gland cell library (S1D and S5 Figs), likely reflect the biology of the system: we speculate that the pre-parasitic J2 nematode cannot express all effectors because it has limited energy reserves; the bulk of the effector repertoire is involved in mediating the processes at the very early stages of infection at 10hpi and 48 hpi, including the transition to biotrophy; once the feeding site is established, a separate, smaller, set of effectors are required for maintenance of the feeding site.

To understand the regulation of this precise control over time, we computed connections to the effector network with transcription factors (TFs) in the parasite genome. In so doing, we inadvertently re-identified the only known regulator of effectors, the subventral gland regulator (sugr) [34]. While sugr is the second most connected transcription factor to the effector network, there are many other transcription factors that are also highly connected. This will be an exciting area of future study because TFs are also strongly associated with most other times of infection, and this method both identifies, and ranks, these transcription factors by their likely impact on the regulation of the effectorome.

Interestingly, the 48 hpi subcluster has no known transcription factors connected to it. There are several possibilities that may explain this observation. One possibility is that the definition of transcription factor used is imperfect. While this is certainly true, it may or may not be the explanation for this conspicuous absence. Another explanation is that consortia of transcription factors are responsible, each of which does not have a sufficiently similar expression profile to cluster with the group, but together produce the observed pattern. Similarly, it is possible that individual isoforms of a transcription factor would have a highly correlated expression with this group, but that when computed on a per-locus basis do not. All of the above assumes that the expression of the transcription factor/factors itself/themselves do indeed change over time. It is certainly possible that transcription factor(s) that control this group are not regulated at the transcriptional level, but are instead regulated by some other post transcriptional/translational mechanism that gives rise to the observed pattern in the effector transcription without a corresponding pattern of transcription of the factor itself. In any case, understanding the regulation of this subcluster, and indeed the other subclusters, will be the focus of future research.

### Evolution

The effectorome is assembled from a diversity of genetic capital that itself evolved over a very long period of time—19% of effector sequences are similar to genes that predate the phylum Nematoda. Therefore, caution is advised when analysing effector identification pipelines that exclude genes similar to those in non-parasitic ancestors (i.e. *Caenorhabditis elegans*): if applied to this species they would have missed almost one in five effectors, including the 2nd and 5th largest families. Generally speaking, dorsal gland effectors tend to be assembled from newer genetic capital, and subventral gland effectors from older genetic capital, although there are many exceptions. The “novel” effectors tend to be assembled from even newer genetic capital (S1 Table), which makes sense because many of the known effectors are identified by homology to effectors in another species and so are by definition at least to some degree conserved. Novels are identified by direct gland cell sequencing and so their identification is not biassed in this way.

A structural similarity network of predicted effectors did not identify sequence unrelated but structurally similar effectors (S3 Fig). This is contrary to the emerging theme (e.g. MAX effectors in ascomycete fungal pathogens [16]) and suggests that, for *H*. *schachtii* effectors at least, protein sequence homology captures the dominant majority of relatedness within the effectorome for those that are foldable today with AlphaFold (44%).

Taking a comparative approach, it will be possible to identify a “core” effectorome of the last common biotrophic ancestor of the cyst nematodes, based on these data. While this will require the complete effectorome of at least one other species, carefully selected for its/their position in the phylum, a rough proxy is presented herein based on available genomic data. Here, if an effector had a similar sequence that is also predicted to be secreted in the cyst nematodes, it was considered to be an effector in said species. Using this information, we can roughly date the emergence of the effector families, and find that many extant families, including the largest, were probably already present in the last common biotrophic ancestor of the cyst nematodes. The conserved ability of the last common biotrophic ancestor to manipulate plant development, metabolism, and physiology (as reviewed by Molloy et al. [6]) likely resides in extant members of this “core” effectorome.

Intuitively, the older effector families also tend to be the larger effector families. Taken together with the highly skewed membership of effector families, this suggests that the effector repertoire has been moulded by large scale re-shaping/expansion of the “core”, coupled with recent addition of many new small effector families, presumably concurrent with the changes in host/genotype over the same period.

### Cross-kingdom regulation

We generated a cross-kingdom transcriptional network for nematode effectors and host plant genes to identify functions that are co-regulated throughout infection. This revealed a highly connected network across all infective stages. By highlighting plant genes of interest in the network (e.g. genes of a particular function or pathway), we can interrogate which plant processes may be altered during parasitism, and when in the life cycle this might occur.

Importantly, the cross-kingdom network differs from the effector-only network in one key aspect: 25% of the connections are strongly negative (0% of the effector-only network were negative connections). This could possibly reflect suppressive interactions between effectors and plant genes, which is consistent with the fact that nematode effectors have been shown to suppress plant immune responses [4,5]. Plant genes with functions in immunity or defence are enriched in the early stages of infection, but the majority of these correlations are positive. This might suggest an initial immune response to the nematode which is later suppressed by nematode effectors. However, we do not know whether the nematodes sampled at 10 hpi were about to successfully infect the plant or not. It is possible that many of these nematodes were unable to overcome the plant immune system, and hence were unable to establish parasitism. While it is tempting to use these data to infer function, this should be tested experimentally before conclusions are drawn.

In contrast, nematodes sampled at the 12 dpi and 24 dpi life-stages are by definition successful, so here we can more confidently hypothesise about roles for effector-plant gene correlations in parasitism. In addition to altering the plant immune system, nematodes also take advantage of plant developmental plasticity to reprogramme many elements of development, physiology and cell biology [6]. Where an effector is correlated with genes involved in plant developmental processes in these parasitic clusters, this effector can be a candidate for development altering functions. Uncovering the development altering “toolbox” of plant-parasitic nematodes can enable biotechnology, crop protection, and uncover fundamental aspects of plant biology. This cross-kingdom transcriptional network can provide a basis for identifying potential targets for future functional work in plant-parasitic nematode effector biology.

## Materials and methods

### Gland cell extraction and sequencing

For the parasitic J2 library generation, gland cells were extracted and library construction proceeded according to previously established methodology for fixed gland cells [39]. For the pre-parasitic J2 and parasitic J3 library generation, non-fixed gland cells were used in library construction. This was done to improve RNA quality, reduce the number of gland cells needed for input, and reduce the overall rRNA contamination in the final library.

In brief, for each biological replication of each life-stage, nematodes of each life-stage were collected using established methods [24]. 50 μl packed volume of each life-stage were washed in 10 mM MES buffered (pH 6.5) water and resuspended in 100 μl of ice cold 3xHank’s Balanced Salts Solution (14065–056 Gibco-BRL), supplemented with 2% Foetal Bovine Serum (A3160601 Gibco-BRL) and 1 U/μl Superase-in RNAse inhibitor (AM2694 ThermoFisher). 35 μl of this nematode suspension was transferred into a RNAseZap treated 60 mm glass petri dish and cut with a vibrating razor blade, with the goal of 2–3 cuts per nematode. The cut nematode pieces were recovered by washing the glass dish with 1 ml ice cold Cutting Buffer and transferring this suspension to a 15 ml conical bottom tube on ice. This was repeated until all of the nematode suspension was cut. The contents of the 15 ml conical bottom tube was filtered through a 25 μm tissue filter (Milintyl Biomacs) and into a new 15 ml conical bottom tube, on ice. The cell filtrate was gently pelleted at 1000 g for 3 minutes with a gentle brake. The supernatant above the cell pellet was removed to approximately 100 μl, DAPI was added to a 1:1000 dilution and this was kept on ice. 30 μl of this suspension was transferred onto a coverglass thickness slide, spread across the slide, gently, with a pipette tip and observed and manipulated under an inverted fluorescent microscope with a micromanipulator attached. Using a microinjection needle with a diameter of approximately 20 μm, we microaspirated a total of 10 gland cells (5 dorsal and 5 subventral pairs) into the needle (use fluorescence and DAPI filter to aid in observing gland cells, if needed) utilising CellTram Oil to generate a vacuum. After the completion of the collection of each biological replication, the set of collected gland cells were transferred into a 5 μl drop of IDTE (10 mM Tris, 0.1 mM EDTA) buffer that has been placed in the neck of a 200 μl thin wall PCR tube laid on its side and placed on a fresh coverglass thickness slide. The micromanipulator and CellTram Oil were used to generate back pressure to expel the gland cells from the needle and into the 5 μl drop. This drop was then spun to the bottom of the tube via a tabletop microcentrifuge, flash frozen and placed at -80°C.

Once all biological replications of glands from each life-stage were collected, the 5 μl samples were used as input into the SMART-Seq v4 Ultra Low Input RNA Kit (Takara Bio USA) (for parasitic J3 library generation) or the SMART-Seq mRNA LP Kit (Takara Bio USA) (for preparasitic J2 library generation). We followed the protocol for starting with RNA or Cells Sorted into Non-CSS Buffer. Additionally, we modified the overall protocol to include a cell lysis optimization step prior to First Strand Synthesis, where we performed 3 rounds of freeze-thaw on all samples to improve cell lysis efficiency. Libraries were sequenced using 150 bp paired end reads.

All RNAseq reads were analysed with FastQC v.0.11.8 [40] and trimmed using BBduk v38.34 (https://github.com/BioInfoTools/BBMap/blob/master/sh/bbduk.sh). Only reads with a minimum Phred Quality Score of 20, minimum length of 75 bp, and without adapters were retained. Low quality bases were also removed from the 5’ ends of reads in accordance with FastQC per base sequence quality analysis. Trimmed reads from each library were mapped to the *H*. *schachtii* 1.2 reference genome [25] using STAR v2.7.10b [41]. Mapped reads were visualised using Apollo [30]. The htseq-count function of HTseq v0.12.4 [42] was used to count read coverage per gene. For the ppJ2 and pJ3 libraries only, uniquely mapped spliced reads were counted to remove artefacts attributed to the low input library prep method for these two gland cell types. Count tables were loaded into R v4.2.1 using the tidyverse package [43] and normalised using the NormalizeTPM function of the R package ADImpute [44]. The clustering of gene counts from normalised RNA-seq. data from each biological replicate was visualised by a Principal Component Analysis (PCA) using the plotPCA function of the R package DESeq2 [45].

### Effector identification

For each of the three life-stages (ppJ2, pJ2 and pJ3), mean normalised gland cell expression was calculated for each gene. Gland cell expression values were sorted into ‘bins’ (each bin represents a range of expression values). For each expression bin the enrichment or depletion of 248 predetermined high-confidence *H*. *schachtii* effector genes from Siddique et al. [25] was determined by a hypergeometric test (S1 Fig). The minimum expression level at which effector genes were enriched established the threshold expression level above which genes were considered to be putative effectors provided they: i) encode predicted secretion signals (SignalP v4.1); ii) contain no TM domains (TMHMM) or iii) ER retention motifs (Regular Expression) [25]. The size of expression bins was chosen individually for each life-stage based on the ‘hit-rate’ (i.e. the ratio of known effector genes to the total number of genes captured above the threshold) with lower ratios being preferred. Gene models for 725 genes encoding known effectors and predicted secreted proteins above the respective thresholds for each life-stage were manually inspected and re-annotated on Apollo [30] where the gene prediction had failed to correctly capture gene structure. For corrected genes, secretion signals and transmembrane domains were re-predicted using SignalP v4.1 [46] and TMHMM [47]. Predetermined “known” effector genes [25] and “novel” highly gland cell expressed predicted effector genes were combined to form a more comprehensive list of 717 predicted *H*. *schachtii* effector genes (S1 Table).

### *In situ* hybridisation

*In situ* hybridisations were performed using ppJ2 of *H*. *schachtii* following previously published methodology [48]. Specific primers were designed to amplify a product for each of the candidate effector genes using a cDNA library produced from ppJ2s (S2 Table). The resulting PCR products were then used as a template for generation of sense and antisense DIG-labelled probes using a DIG-nucleotide labelling kit (Roche, Indianapolis, IN, USA). Hybridised probes within the nematode tissues were detected using an anti-DIG antibody conjugated to alkaline phosphatase and its substrate. Nematode segments were observed using a DP73 digital Olympus camera mounted on a Bx51 Olympus microscope.

### Effector family prediction

Orthogroups were assigned to predicted effector genes based on a pre-computed OrthoMCL analysis including 59 species across the phylum Nematoda, and 2 outgroup Tardigrade species [32]. Previously, 248 high-confidence effectors were assigned to effector families based on sequence similarity to known plant-parasitic nematode effectors, and the presence of known effector motifs [25]. Novel predicted effectors which clustered into the same orthogroups, or shared key functional annotations (Pfam domains) with a known effector (e.g. glutathione synthetase (GS)-like domains [31]) were considered to be in the same effector family. For putative effectors which did not contain characteristic effector Pfam dominas, or share an orthogroup with a known effector, orthogroups were used to define predicted families. Genes with no informative Pfam domains and no assigned orthogroup were compared by sequence similarity (BLAST) to all effector genes. All BLAST alignments with an e-value above 1 x 10^−5^ were manually inspected and families were assigned accordingly. Expert knowledge was used to assign predicted effector genes to families where the nature of the effector precludes identification by Pfam domains or sequence similarity (e.g. CLEs [33] do not have Pfam domains and their short size means they do not function well in BLAST-based analyses). After these combined analyses, genes with no assigned family were considered to be *H*. *schachtii* specific ‘orphans’. Orphans with sequence similarity to other orphans were assigned to ‘orphan families’.

### Evolutionary origins

Evolutionary origins of predicted effectors were assigned based on pre-computed OrthoMCL data [32]. Sequences with orthologs in other nematode (or tardigrade outgroup) species were considered to have been present in the last common ancestor shared between that species and *H*. *schachtii*. Assigned evolutionary origins were then manually curated and updated where expert knowledge contradicted OrthoMCL data or data was absent (e.g. where a GS domain is present, ‘predates nematodes’ was assigned as the sequence origin because the sequence that gave rise to GS effectors predates the phylum Nematoda [31]). Orthogroups containing putative *H*. *schachtii* effectors were identified and corresponding members of those orthogroups from either *G*. *pallida* [49,50] or *G*. *rostochiensis* [19] were analysed for the presence of secreted proteins. The presence of a secreted ortholog in one of these species was used as a rough proxy for the presence of a homologous effector in the last common ancestor of cyst nematodes.

### Transcriptional network analyses

Expression profiles of predicted effectors across the nematode life cycle [25] were loaded into R v4.2.1 using the tidyverse package [43]), and pairwise distance correlation coefficients were computed using the energy R package (https://github.com/mariarizzo/energy). A correlation matrix of distance correlation coefficients between genes was generated at an arbitrary edge threshold of 0.975. Various attributes were assigned to nodes in the network (expression supercluster [25], gland cell expression (S1 Table, assembled from the literature and *in situ* hybridisations in this paper), and evolutionary pressure (described above), using custom R scripts (all relevant scripts are available at: https://github.com/BethMolloy/Effectorome_H_schachtii). Directionality of correlation was estimated using Pearson’s correlation coefficient and added as an edge attribute to the network. Transcriptional network files are deposited in DRYAD accession DOI: 10.5061/dryad.rfj6q57hn [51]. The *H*.*schachtii* TFome prediction was based on the Pfam domains found in the *C*. *elegans* TFome as defined by Kummerfeld and Teichmann (DBD database, [52]) and Hu et al. (AnimalTFDB v3.0, [53]) with the addition of PF00105 (Zinc finger, C4 type (two domains)). Predicted transcription factors (TFs) with expression in at least two gland cell libraries were added to the effector network if they shared at least one connection with an effector (above a threshold distance correlation coefficient of 0.975). The number of connections with predicted effectors for each TF was added as a node attribute and used to determine height in the Z axis. All networks were visualised using Gephi v0.10.1 [54].

For the cross-kingdom transcriptional network, mean uninfected sample expression values were subtracted from infected sample expression values to isolate infection-specific changes in gene expression [25]. Distance correlation coefficients were computed between the expression profiles of effector genes and normalised plant genes during the five infective life-stages. Plant genes were included in the network if they were successfully assigned to a supercluster by Siddique et al. [25] and shared at least one connection with an effector (above a threshold distance correlation coefficient of 0.975). Likewise, effector-effector connections were also included in the network, while effector genes with no connections to any other effectors or plant genes were excluded to aid visualisation.

*Arabidopsis thaliana* immunity/defence-related genes were defined as: genes annotated with a GO term in both the GO Slim categories “response to biotic stimulus” (GO:0009607) and “response to stress” (GO:0006950) (according to GO Slim Classification for Plants [38]); or genes annotated with the GO term “response to wounding” (GO:0009611); or genes annotated with any GO term containing the phrases “defence response” or “immune”. Presence or absence of each plant in this immunity/defence dataset was then added as an attribute to the network.

Enrichment or depletion of connections between immunity/defence-related genes and effectors in a given supercluster (as defined by Siddique et al. [25]) was calculated using a bootstrapping approach. The total number of connections to immunity/defence-related plant genes was counted for each effector supercluster and compared to 1,000 simulations in which immunity/defence-related gene identity was assigned at random to plant genes in the network using R function Sample without replacement. Where the true number of immunity/defence-related genes connected to a given supercluster was greater or less than 95% confidence intervals expected for random assignment of immunity/defence-related genes, immunity/defence-related genes were considered enriched or depleted. All relevant scripts are available at: at: https://github.com/BethMolloy/Effectorome_H_schachtii.

### Protein structure prediction and clustering

Signal peptides were cleaved from amino acid sequences for all secreted proteins in the *H*. *schachtii* [25], and *G*. *rostochiensis* genomes [19] using SignalP v4.1. Sequences were aligned to the ColabFold v1.5.2 [55,56] database using build in MMseqs2 [57]. Where effectors were manually annotated and corrected, the corrected sequences were used in place of the original gene predictions. Protein structure was predicted using ColabFold v1.5.2 [55,56] which has AlphaFold v2.3.1 integrated, with three recycles per model. Predicted folds from the genome with an average pLDDT score < 50 and a pTM score of < 0.5, were discarded. Predicted structures are deposited in DRYAD accession DOI: 10.5061/dryad.rfj6q57hn. Structural similarity between effectors was predicted by an all-vs-all search using Foldseek [58,59] and connections in the similarity network were permitted at TM-scores of >0.5 and above. Relevant scripts are available at: https://github.com/BethMolloy/Effectorome_H_schachtii/tree/main/ProteinFolding.

### Dryad DOI

DOI: 10.5061/dryad.rfj6q57hn [51]

## Supporting information

S1 Fig Gland cell enrichment of effectors and putatively secreted proteins. **A-C)** Effector enrichment in gland cell libraries. Putative effectors were identified using enrichment of effector-annotated genes and putative secreted proteins to identify an expression cutoff above which putatively secreted proteins are likely effectors. The upper axis shows the total number of genes (left) and the number of effector-annotated genes and putative secreted proteins (right) in each expression bin (i.e. at each expression level). Hypergeometric distribution tests were used to determine either the enrichment (green arrows) or depletion (red arrows) of effectors or secreted proteins in each bin. The lower axis shows the p-values from these tests. The horizontal dashed line denotes a p-value of 0.001. The vertical dashed line denotes the threshold expression level above which effector genes and or secreted proteins are largely or consistently enriched. **F**) Proportional Venn-diagram showing which gland cell libraries from which putative effectors were identified.
(TIF)

S2 Fig Transcriptional network of a random set of 717 *Heterodera schachtii* genes. A transcriptional network of a random set of 717 *H*. *schachtii* genes. Each circle represents one locus, and connections between circles indicate a correlation in expression of 0.975 or above (distance correlation coefficient) across the life cycle. The key indicates the expression supercluster as defined by Siddique et al. [25]—where, for example, genes with expression peaking at J2 are shown in red, 10 hours post infection in yellow, and J2_10 hours post infection shown in orange.
(TIF)

S3 Fig Structural similarity network for folded putative effectors. A structural similarity network for the predicted structures of putative *H*. *schachtii* effectors. Each circle represents one foldable effector gene locus (i.e. a fold with an average pLDDT > 50 and pTM > 0.5), and connections between circles indicate structural similarity TM-score of 0.5 or above as determined using structure-based BLAST, Foldseek [37]). Colours indicate effector families as assigned in this study (S1 Table).
(TIF)

S4 Fig Hybrid CBP/Expansin effector identified by structural similarity of folded putative effectors. Predicted structures of *H*. *schachtii* effectors, showing **A)** an effector with an Expansin domain only, **B)** a hybrid effector with both a cellulose binding protein (CBP) domain and an Expansin domain, and **C)** an effector with a CBP domain only. Protein structures were predicted using ColabFold (using AlphaFold). **D)** An amino acid alignment of the three folded effectors. **E)** The superposed structures of expansin and CBP effectors onto hybrid CBP/Expansin effector.
(TIF)

S5 Fig Effector enrichment analysis by library. Putative effectors were identified using enrichment of effector-annotated genes and putative secreted proteins to identify an expression cutoff above which putatively secreted proteins are likely effectors. For each of the pre-parasitic J2 (yellow), parasitic J2 (orange), and parasitic J3 (red) life-stages, the effectors that were identified are mapped to the network.
(TIF)

S1 Table The putative effectorome of the plant-parasitic cyst nematode *Heterodera schachtii*. (XLSX)

S2 Table Primers used for *in situ* hybridisation of putative *Heterodera schachtii* effector gene transcripts. (XLSX)
