# Supplementary figures and images for: The origin, deployment, and evolution of a plant-parasitic nematode effectorome
Source: PLoS Pathog. 2024 Jul 29;20(7):e1012395. doi: 10.1371/journal.ppat.1012395 (PMC11309470; doi:10.1371/journal.ppat.1012395)

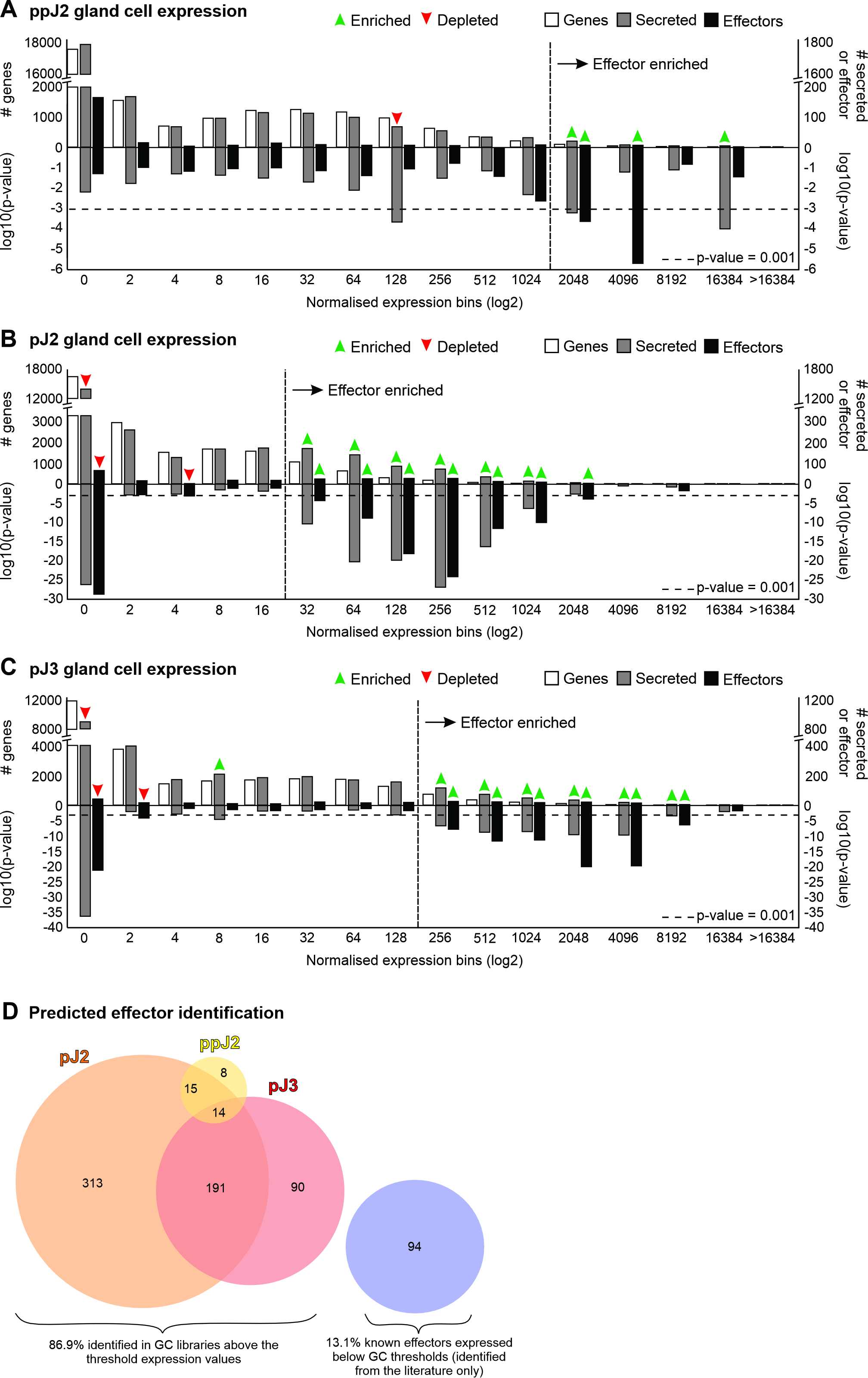

Supplement: S1 Fig — A-C) Effector enrichment in gland cell libraries. Putative effectors were identified using enrichment of effector-annotated genes and putative secreted proteins to identify an expression cutoff above which putatively secreted proteins are likely effectors. The upper axis shows the total number of genes (left) and the number of effector-annotated genes and putative secreted proteins (right) in each expression bin (i.e. at each expression level). Hypergeometric distribution tests were used to determine either the enrichment (green arrows) or depletion (red arrows) of effectors or secreted proteins in each bin. The lower axis shows the p-values from these tests. The horizontal dashed line denotes a p-value of 0.001. The vertical dashed line denotes the threshold expression level above which effector genes and or secreted proteins are largely or consistently enriched. F) Proportional Venn-diagram showing which gland cell libraries from which putative effectors were identified. (TIF) [file ppat.1012395.s001.tif]

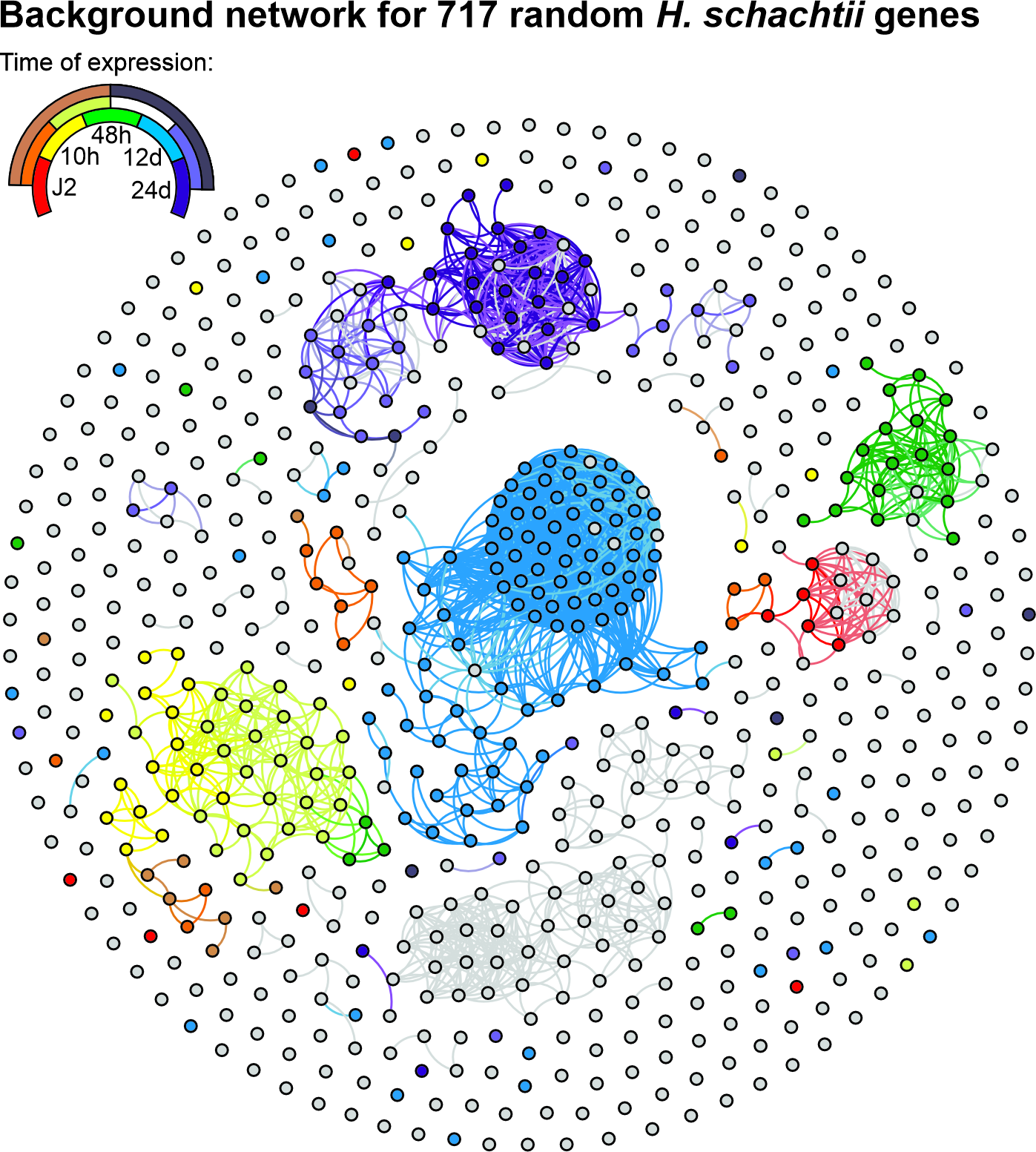

Supplement: S2 Fig — A transcriptional network of a random set of 717 H. schachtii genes. Each circle represents one locus, and connections between circles indicate a correlation in expression of 0.975 or above (distance correlation coefficient) across the life cycle. The key indicates the expression supercluster as defined by Siddique et al. [25]—where, for example, genes with expression peaking at J2 are shown in red, 10 hours post infection in yellow, and J2_10 hours post infection shown in orange. (TIF) [file ppat.1012395.s002.tif]

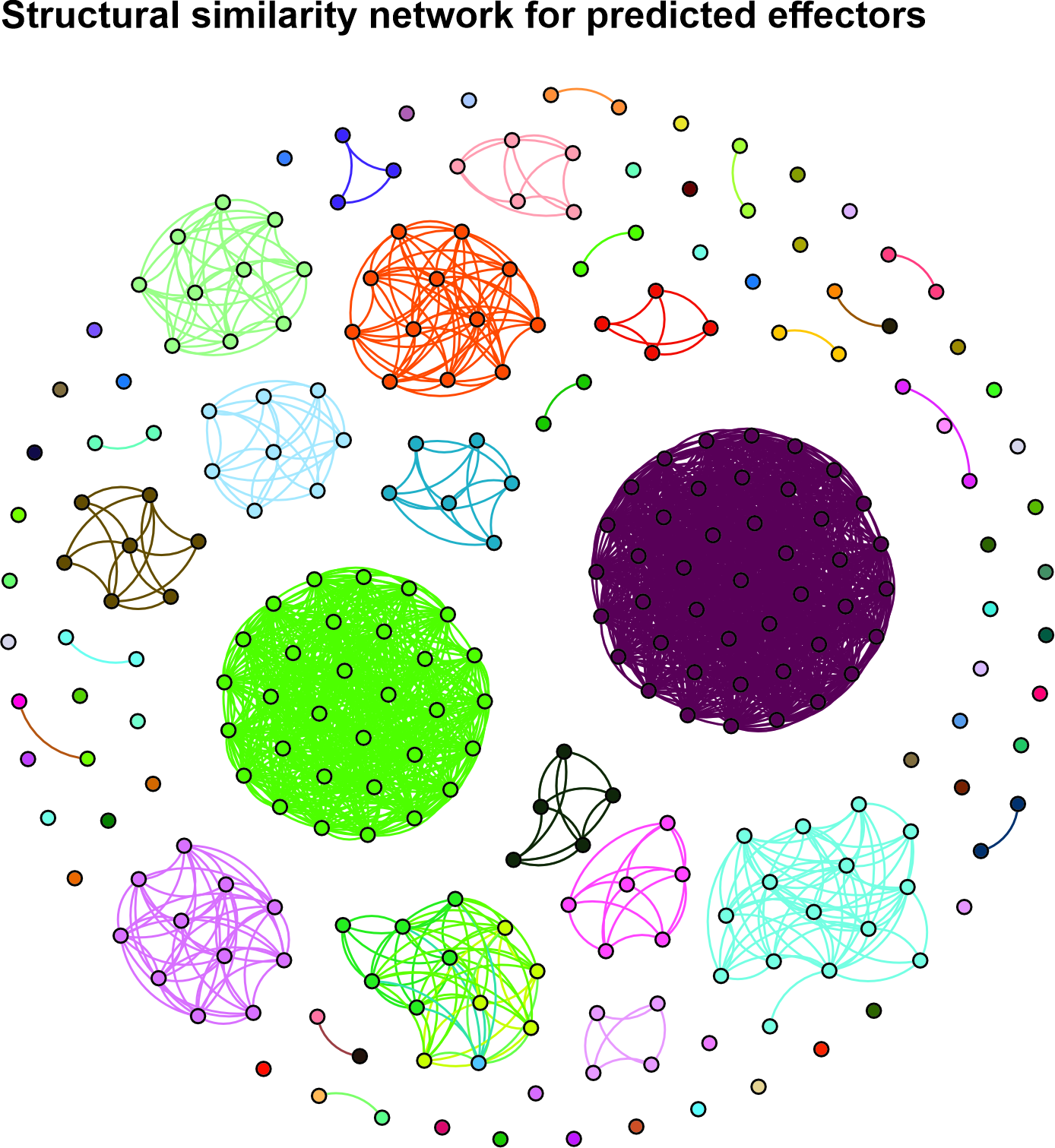

Supplement: S3 Fig — A structural similarity network for the predicted structures of putative H. schachtii effectors. Each circle represents one foldable effector gene locus (i.e. a fold with an average pLDDT > 50 and pTM > 0.5), and connections between circles indicate structural similarity TM-score of 0.5 or above as determined using structure-based BLAST, Foldseek [37]). Colours indicate effector families as assigned in this study (S1 Table). (TIF) [file ppat.1012395.s003.tif]

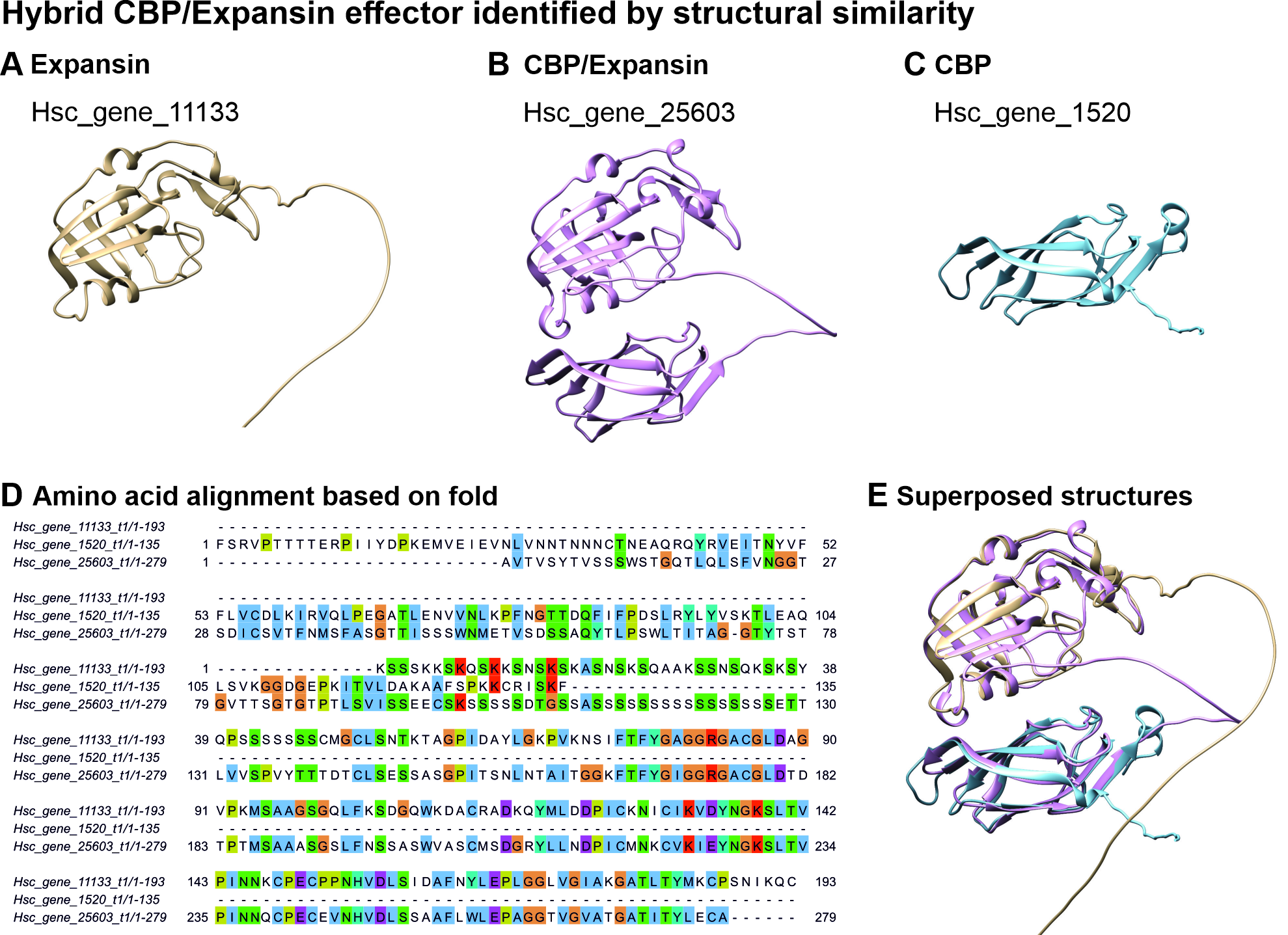

Supplement: S4 Fig — Predicted structures of H. schachtii effectors, showing A) an effector with an Expansin domain only, B) a hybrid effector with both a cellulose binding protein (CBP) domain and an Expansin domain, and C) an effector with a CBP domain only. Protein structures were predicted using ColabFold (using AlphaFold). D) An amino acid alignment of the three folded effectors. E) The superposed structures of expansin and CBP effectors onto hybrid CBP/Expansin effector. (TIF) [file ppat.1012395.s004.tif]

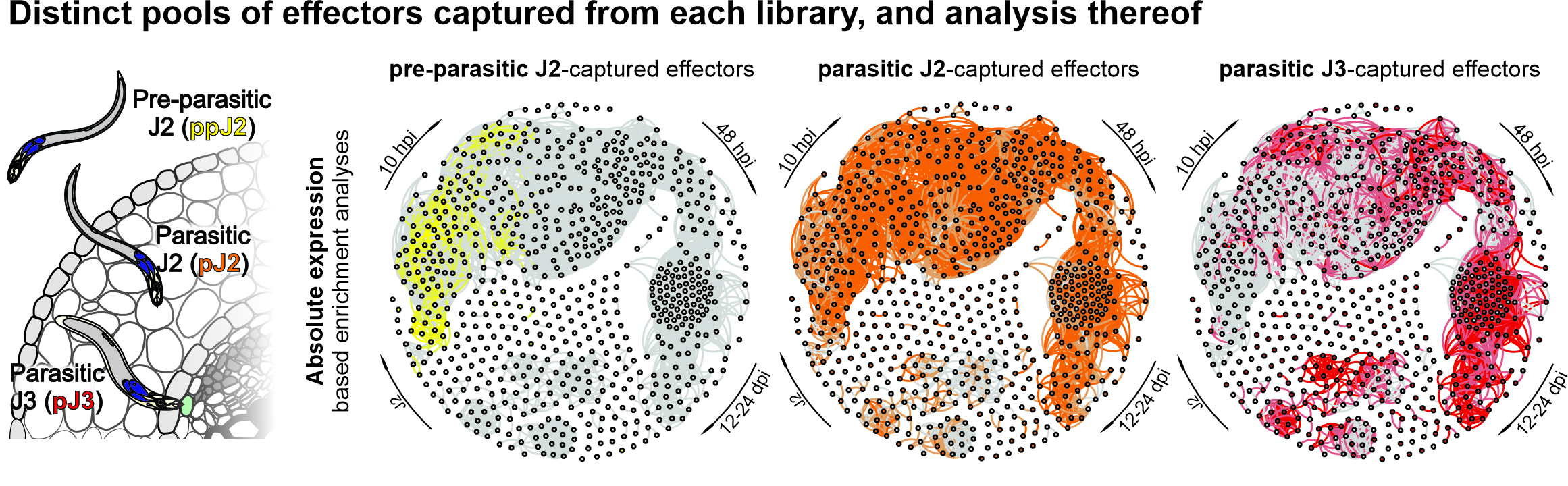

Supplement: S5 Fig — Putative effectors were identified using enrichment of effector-annotated genes and putative secreted proteins to identify an expression cutoff above which putatively secreted proteins are likely effectors. For each of the pre-parasitic J2 (yellow), parasitic J2 (orange), and parasitic J3 (red) life-stages, the effectors that were identified are mapped to the network. (TIF) [file ppat.1012395.s005.tif]
